# Differential gene expression along the animal-vegetal axis in the ascidian embryo is maintained by a dual functional protein Foxd

**DOI:** 10.1371/journal.pgen.1006741

**Published:** 2017-05-17

**Authors:** Shin-ichi Tokuhiro, Miki Tokuoka, Kenji Kobayashi, Atsushi Kubo, Izumi Oda-Ishii, Yutaka Satou

**Affiliations:** Department of Zoology, Graduate School of Science, Kyoto University, Kyoto, Japan; New York University, UNITED STATES

## Abstract

In many animal embryos, a specific gene expression pattern is established along the animal-vegetal axis soon after zygotic transcription begins. In the embryo of the ascidian *Ciona intestinalis*, soon after the division that separates animal and vegetal hemispheres into distinct blastomeres, maternal Gata.a and β-catenin activate specific genes in the animal and vegetal blastomeres, respectively. On the basis of these initial distinct gene expression patterns, gene regulatory networks promote animal cells to become ectodermal tissues and vegetal cells to become endomesodermal tissues and a part of the nerve cord. In the vegetal hemisphere, β-catenin directly activates *Foxd*, an essential transcription factor gene for specifying endomesodermal fates. In the present study, we found that *Foxd* also represses the expression of genes that are activated specifically in the animal hemisphere, including *Dmrt1*, *Prdm1-r*.*a* (*Bz1*), *Prdm1-r*.*b* (*Bz2*), and *Otx*. A reporter assay showed that *Dmrt1* expression was directly repressed by Foxd, and a chromatin immunoprecipitation assay showed that Foxd was bound to the upstream regions of *Dmrt1*, *Prdm1-r*.*a*, *Prdm1-r*.*b*, and *Otx*. Thus, Foxd has a dual function of activating specific gene expression in the vegetal hemisphere and of repressing the expression of genes that are normally expressed in the animal hemisphere. This dual function stabilizes the initial patterning along the animal-vegetal axis by β-catenin and Gata.a.

## Author summary

In embryogenesis of most animals, a specific gene expression pattern is established along the animal-vegetal axis first. In the embryo of the ascidian *Ciona intestinalis*, the activity of the maternal factor Gata.a is suppressed by β-catenin, which is active only in the vegetal hemisphere, and thereby these two factors activate specific genes in the animal and vegetal blastomeres, respectively. We found that a gene encoding a transcription factor, Foxd, which is a direct target of β-catenin, works as a promoter for endomesodermal fate and an inhibitor for ectodermal fate. In the ascidian embryo, the animal-vegetal axis initially established by the maternal factors is not stable enough for subsequent developmental processes, and needs to be maintained by Foxd. Thus, the animal hemisphere fate is suppressed first by the maternal factor β-catenin, and then by Foxd, which is activated by β-catenin. The primary embryonic axis is not stable initially, and stabilized by a transcription factor, which is expressed differentially along the axis.

## Introduction

In many animal embryos, localized maternal factors create differential gene expression patterns along the animal-vegetal axis [1–3], and the subsequent developmental program proceeds on the basis of this initial patterning. In ascidian unfertilized eggs, several identified and unidentified maternal factors are unequally distributed along the animal-vegetal axis [4]. At the 8-cell stage, the animal and vegetal hemispheres become separated into distinct blastomeres, and the difference along the animal-vegetal axis is clearly established; when blastomeres are experimentally isolated at the 8-cell stage, endomesodermal cells are differentiated from vegetal hemisphere cells [5–8], and epidermal cells are differentiated from animal cells [9]. In 16-cell embryos of the ascidian *Ciona intestinalis*, the maternal transcription factor Gata.a activates *Ephrina*.*d* and *Tfap2-r*.*b* specifically in the animal hemisphere, and a complex of β-catenin and Tcf7 activates *Foxd* and *Fgf9/16/20* in the vegetal hemisphere [10–15]. In the vegetal hemisphere, β-catenin/Tcf7 weakens the Gata.a-binding activity for target sites through a physical interaction, and thereby the animal hemisphere genes are not expressed in the vegetal hemisphere at the 16-cell stage [15]. In this manner, the initial difference between the animal and vegetal hemispheres is set up.

*Foxd* and *Fgf9/16/20*, which are activated by β-catenin/Tcf7, encode a transcription factor and a signaling molecule, respectively. These molecules are required for expression of endodermal and mesodermal genes including *Lhx3/4*, *Zic-r*.*b* (*ZicL*), and *Brachyury* in the vegetal hemisphere [16–18]. In addition, Fgf9/16/20 signaling also induces expression of neural genes including *Dmrt1*, *Otx*, *Prdm1-r*.*a* and *Prdm1-r*.*b* in the neural lineage of the animal hemisphere [11, 19–22]. Animal hemisphere cells that are not induced by Fgf9/16/20 signaling give rise to epidermal cells under the control of *Tfap2-r*.*b*, which encodes a transcription factor [23]. Thus, the difference between the animal and vegetal hemispheres are critically important for subsequent developmental programs.

However, the initial difference between the animal and vegetal hemispheres, which is established by Gata.a and β-catenin/Tcf7, may not be sufficient for explaining differential gene expression patterns between them at the 32-cell stage and thereafter, because two animal hemisphere genes *Dmrt1* and *Dlx*.*b* are expressed ectopically in the vegetal hemisphere of *Foxd* morphants at the early gastrula stage [19]. *Dmrt1* is important for anterior neural and palp (a placode-like structure) fate specification [19, 24], and *Dlx*.*b* is important for neural and epidermal fate specification [23]. In the present study, we examined how the animal-vegetal axis is maintained at the 32-cell stage and thereafter, and showed that *Foxd* acts as a robust binary switch to stabilize the initial patterning along the animal-vegetal axis by Gata.a and β-catenin/Tcf7.

## Results

### Candidate genes under the control of *Foxd* in early embryos

**Fig 1:**
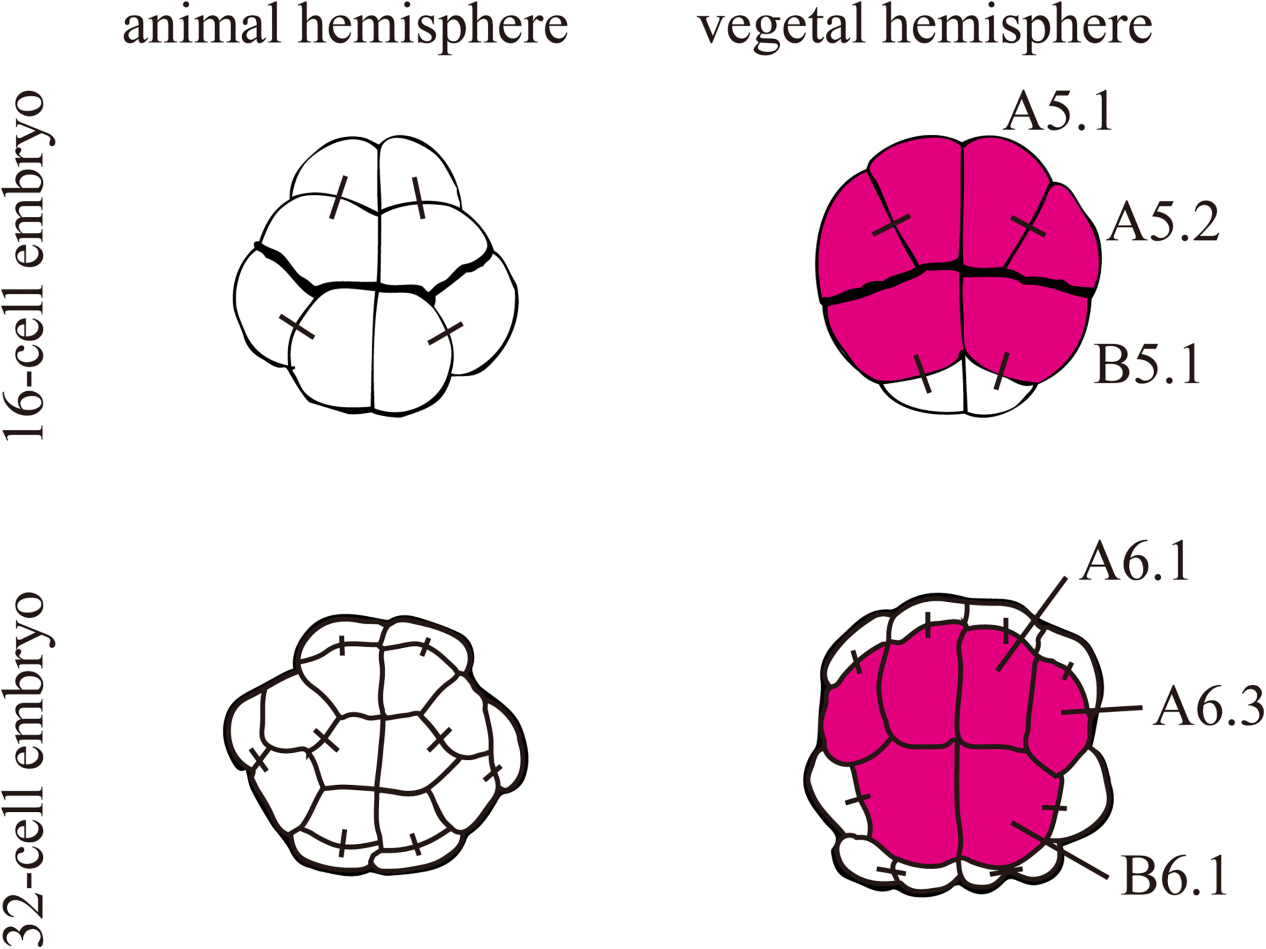
Expression of *Foxd* at the 16- and 32-cell stages. Schematics of the animal and vegetal hemispheres of the bilaterally symmetrical 16-cell and 32-cell embryos. Cells expressing *Foxd* are colored in magenta. Their blastomere names are indicated in the right halves. Note that *Foxd* is expressed only at the 16-cell and 32-cell stages in early embryos. Black bars connecting two cells indicate their sister cell relationship.

**Fig 2:**
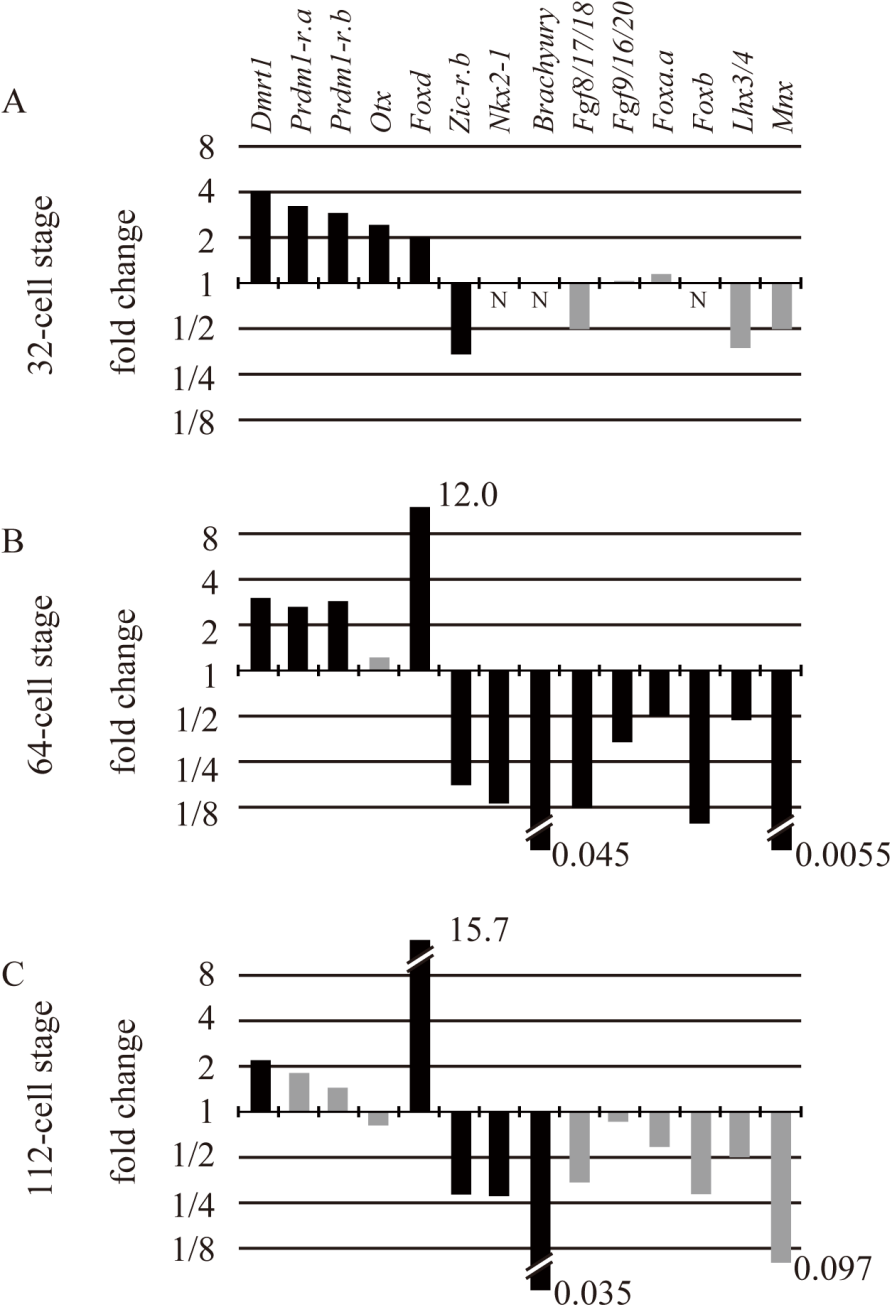
Differential gene expression upon *Foxd* knockdown revealed by RNA-seq analysis. (A–C) Among regulatory genes that are expressed zygotically in early embryos, genes greatly upregulated or downregulated in morphant embryos of *Foxd* are shown (black bars, q-values in NOIseq-sim > 0.80; gray bars, q-values in NOIseq-sim < 0.80). No bars are shown for genes in which no sequence tags were detected (N). The analyses were performed at the 32-cell (A), 64-cell (B), and 112-cell stages (C).

*Foxd* is expressed under the direct control of β-catenin/Tcf7 in three vegetal cell pairs (A5.1, A5.2, and B5.1) of 16-cell embryos (Fig 1). After the next division, among their daughter cells, cells with endodermal fate continue to express *Foxd* (A6.1, A6.3, and B6.1), and the expression becomes undetectable at the 64-cell stage. To identify genes regulated by *Foxd* in early embryos, we performed RNA-seq analysis at the 32-cell, 64-cell, and 112-cell stages to compare transcriptomes between unperturbed and *Foxd* knocked-down embryos. For *Foxd* knockdown, we used a morpholino oligonucleotide (MO) against *Foxd*. We picked up genes encoding transcription factors and signaling molecules that are known to be expressed zygotically between the 32-cell and 112-cell stages [25], and compared expression levels between unperturbed and *Foxd* morphant embryos (Fig 2). We did not utilize biological replicates because we used these data for screening purposes and because we performed this analysis at three successive time points. Fourteen genes were identified to be differentially expressed at one or more stages by a computer program called NOISeq [26] (> 80%, probability of differential expression by NOIseq-sim, which simulates technical replicates). Among them, nine genes were previously known to be regulated by Foxd: *Zic-r*.*b* (*ZicL*), *Brachyury*, *Fgf8/17/18*, *Fgf9/16/20*, *Foxb*, *Lhx3/4*, and *Mnx* were known to be positively regulated by Foxd, and *Dmrt1* and *Foxd* itself are known to be negatively regulated [16, 19, 27]. These observations indicate that the RNA-seq experiments successfully identified genes under the control of *Foxd*.

In addition to these nine previously characterized genes, there were five differentially expressed regulatory genes identified: *Foxa*.*a* was downregulated at the 64-cell stage, *Nkx2-1* (*Ttf1*) was downregulated at the 64-cell and 112-cell stages, *Otx* was upregulated at the 32-cell stage, and *Prdm1-r*.*a* (*Bz1*) and *Prdm1-r*.*b* (*Bz2*) were upregulated at the 32- and 64-cell stages in *Foxd* morphants. These genes were candidates for Foxd targets that had not yet been identified.

### Genes positively regulated by *Foxd* were expressed in the vegetal hemisphere

**Fig 3:**
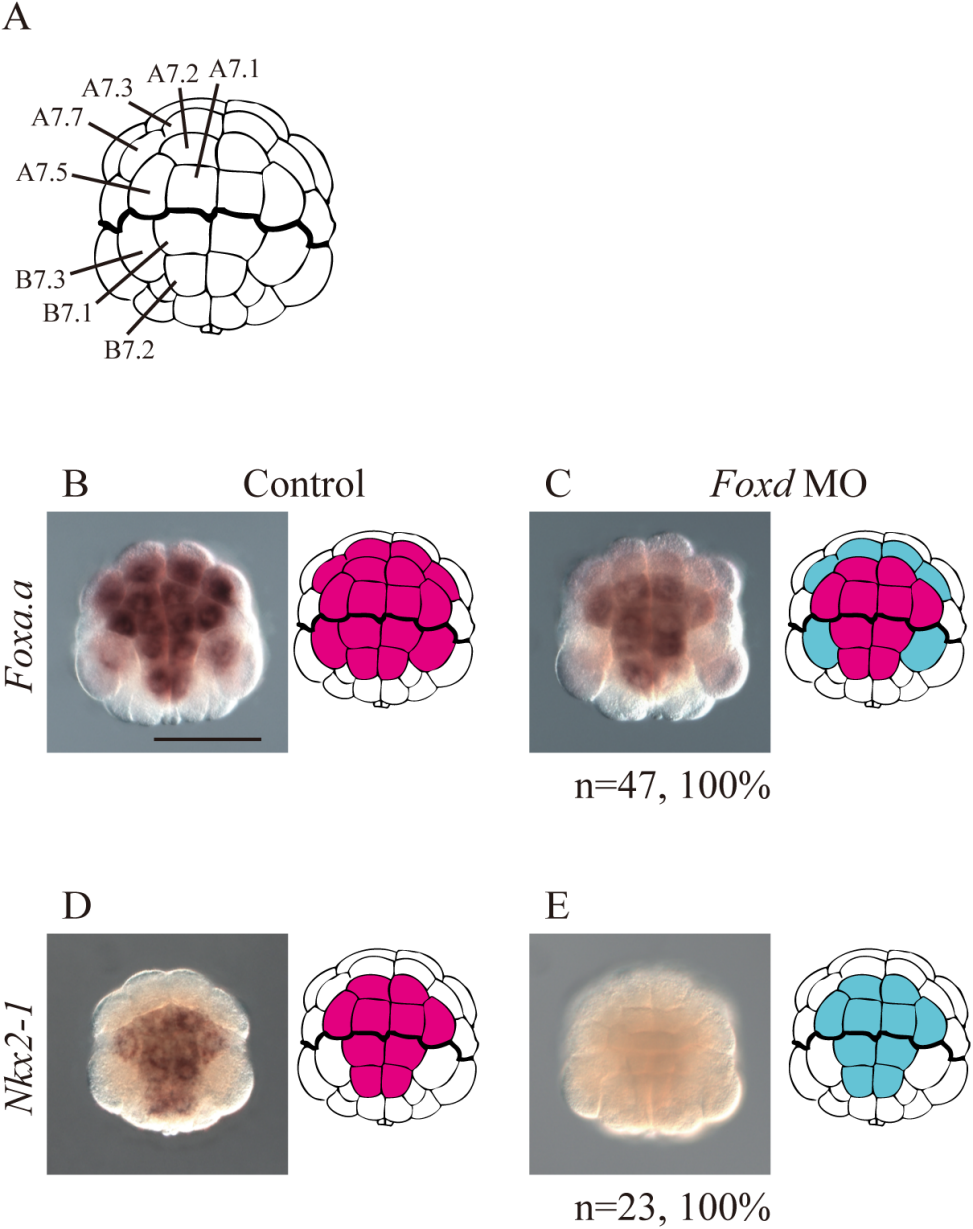
*In situ* hybridization analysis to determine if *Foxa*.*a* and *Nkx2-1* are controlled by Foxd at the 64-cell stage. (A) Illustrations of the vegetal hemisphere of the 64-cell embryo. Blastomere names are indicated in the left half of the bilaterally symmetrical embryo. (B–E) The expression of (B, C) *Foxa*.*a*, and (D, E) *Nkx2-1* in (B, D) control unperturbed embryos and (C, E) *Foxd* morphant embryos. Illustrations on the right indicate the expression patterns. Blastomeres with expression are filled in magenta. Blastomeres that lost expression in *Foxd* morphants are shown in cyan. The number of morphant embryos examined and the proportion of embryos that each panel represents are shown below the panels. Scale bar, 100 μm.

To confirm downregulation of *Foxa*.*a* and *Nkx2-1* in *Foxd* morphant embryos, we performed *in situ* hybridization. *Foxa*.*a* was normally expressed strongly in the vegetal blastomeres designated A7.1, A7.2, A7.3, A7.5, A7.7, B7.1, and B7.2, and weakly in B7.3 at the 64-cell stage (Fig 3A and 3B). *Foxa*.*a* expression was lost only in A7.3, A7.7, and B7.3 in *Ciona Foxd* morphants (Fig 3C). *Foxa*.*a* expression begins at the 8-cell stage, and our data did not indicate downregulation of *Foxa*.*a* at the 32-cell stage (Fig 2A), which is consistent with a recent study [16]. *Nkx2-1* was normally expressed in the vegetal blastomeres designated A7.1, A7.2, A7.5, B7.1, and B7.2 at the 64-cell stage (Fig 3D), whereas it was not expressed in *Foxd* morphants (Fig 3E), as recently shown at the early gastrula stage [16]. Thus, *Foxd* positively regulated *Foxa*.*a* and *Nkx2-1*.

In addition to *Foxa*.*a* and *Nkx2-1*, the genes *Brachyury*, *Fgf8/17/18*, *Fgf9/16/20*, *Foxb*, *Mnx*, and *Zic-r*.*b*, which were found to be positively regulated by Foxd (Fig 2), are all expressed in the vegetal hemisphere [17, 25, 27, 28]. Namely, genes that were identified to be positively regulated by *Foxd* in early embryos were all expressed in the vegetal hemisphere.

### Genes negatively regulated by *Foxd* were expressed in the animal hemisphere

**Fig 4:**
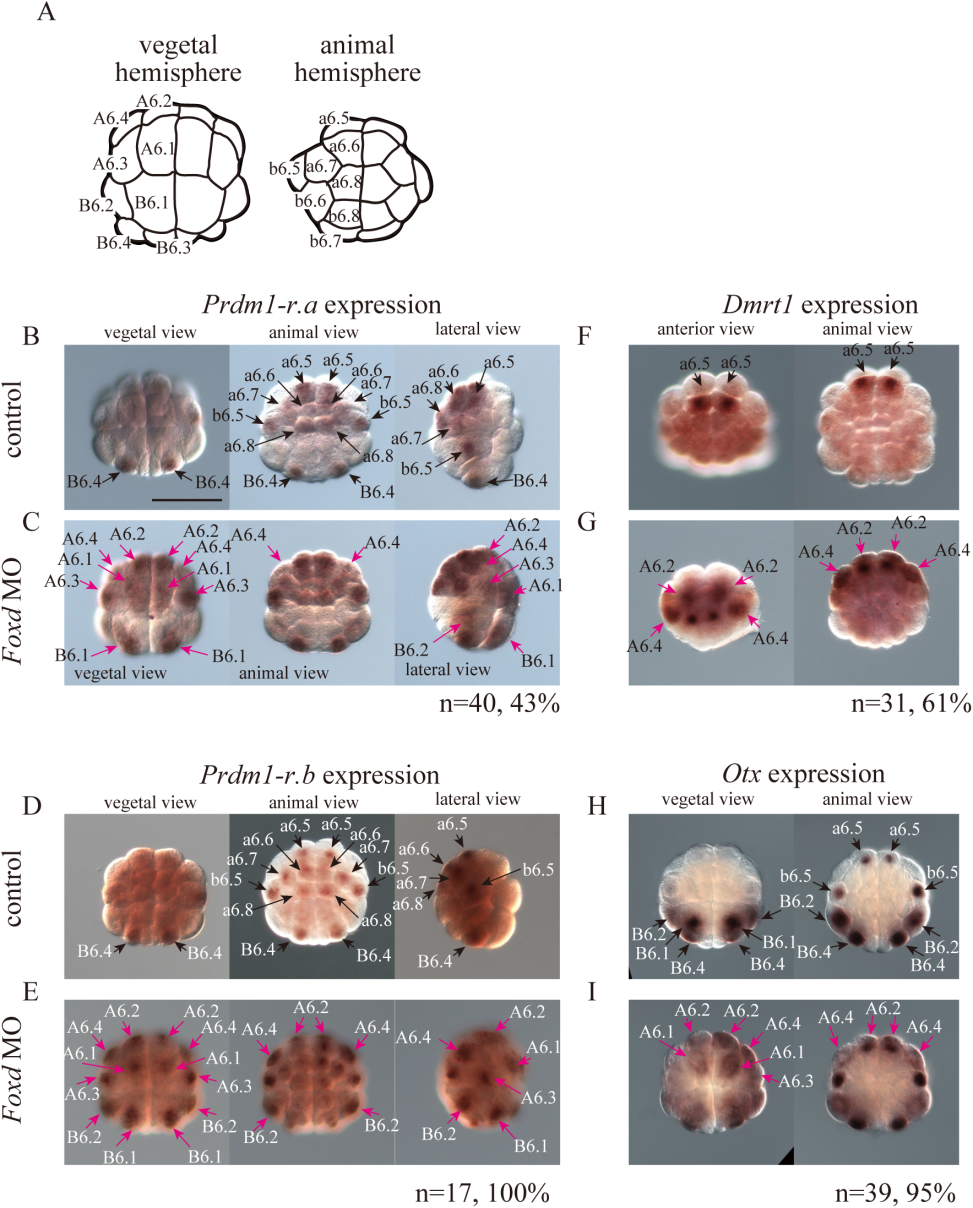
Ectopic expression of animal hemisphere genes in the vegetal hemisphere of *Foxd* morphant embryos at the 32-cell stage. (A) Illustrations of the 32-cell embryo. Blastomere names are indicated in the left half of the bilaterally symmetrical embryo. (B–I) The expression of (B, C) *Prdm1-r*.*a* (*Bz1*), (D, E) *Prdm1-r*.*b* (*Bz2*) (F, G) *Dmrt1*, and (H, I) *Otx* in (B, D, F, H) control unperturbed embryos, and (C, E, G, I) *Foxd* morphant embryos. Black arrows in (B, D, F, H) indicate expression in normal embryos, while magenta arrows in (C, E, G, I) indicate ectopic expression in *Foxd* morphant embryos. The number of morphant embryos examined and the proportion of embryos that each panel represents are shown below the panels. Scale bar, 100 μm. Note that *Fgf9/16/20* is activated independently of *Foxd* at the 16-cell and 32-cell stages (S1D and S1E Fig; Fig 2A) [14–17], although it is later activated by *Foxd* (Fig 2B) [19]. Therefore, it is not strange that *Otx* expression in the animal hemisphere, which is under control of *Fgf9/16/20* [11, 20, 21, 45, 46], was not affected at the 32-cell stage.

*Prdm1-r*.*a*, *Prdm1-r*.*b*, *Foxd*, *Dmrt1*, and *Otx* were found to be negatively regulated by *Foxd* (Fig 2). While *Prdm1-r*.*a* and *Prdm1-r*.*b* are normally expressed in five pairs of animal cells (a6.5 to a6.8 and b6.5) and a pair of vegetal cells (B6.4) at the 32-cell stage [29], these two genes were ectopically expressed in vegetal cells of *Foxd* morphants (A6.1 to A6.4, B6.1, and B6.2) (Fig 4A–4E).

*Foxd* expression was examined in *Foxd* morphants (S1A and S1B Fig). *Foxd* mRNA was detected in *Foxd* morphants at the 64-cell stage, while it was rarely detected in normal 64-cell embryos. This might suggest that Foxd negatively regulates itself, or alternatively, that *Foxd* mRNA was stabilized by binding the MO. To discriminate between these possibilities, we injected synthetic *Foxd* mRNA into *Ciona* eggs. Because the synthetic mRNA lacked the endogenous 3’-UTR, we were able to measure the amount of the endogenous *Foxd* mRNA by RT-qPCR with primers designed to its 3’-UTR. While levels of the maternal control mRNA *Pou2* were unchanged, *Foxd* mRNA levels were greatly reduced by injection of synthetic *Foxd* mRNA (S1C Fig). Therefore, *Foxd* indeed regulates itself negatively.

We previously showed that *Dmrt1* is expressed at the 64- and 112-cell stages in the anterior neural lineage of the animal hemisphere [25], and the RNA-seq result of the present study suggested that this gene was expressed in 32-cell embryos under the control of *Foxd*. Indeed, upon careful re-examination, we detected a weak signal in the anterior animal cells (a6.5) at the 32-cell stage of normal embryos. This expression pattern was expanded to the anterior vegetal cells (A6.2 and A6.4) of *Foxd* morphants (Fig 4F and 4G). Consistently, injection of *Foxd* mRNA reduced *Dmrt1* expression (S1C Fig).

*Otx* is expressed in three pairs of vegetal cells (B6.1, B6.2, and B6.4) and two pairs of animal cells (a6.5 and b6.5) at the 32-cell stage in normal embryos [20]. This gene was expressed ectopically in the anterior vegetal cells (A6.1 to A6.4) of *Foxd* morphants (Fig 4H and 4I).

*Otx* and *Dmrt1* are activated by Fgf signaling [19, 20], and *Fgf9/16/20* is downregulated at later stages in *Foxd* morphants [19], which was consistent with the RNA-seq result at the 64-cell stage (Fig 2B). On the other hand, *Fgf9/16/20* is not downregulated at the 32-cell stage [16, 17], which was also consistent with the RNA-seq result at the 32-cell stage (Fig 2A). Indeed, *Fgf9/16/20* was not downregulated at the 16-cell stage in *Foxd* morphants (S1D and S1E Fig). Therefore, it is likely that the earliest expression of *Fgf9/16/20*, which is controlled by maternal β-catenin [15] but not by Foxd, induced *Otx* and *Dmrt1* expression, even in *Foxd* morphants.

Because *Tfap2-r*.*b* is regulated directly by a maternal factor [15] and expressed in the animal hemisphere at the 16-cell stage [25], and because expression of *Tfap2-r*.*b* was not significantly changed in our RNA-seq experiment (~1.7 fold-increase), we examined the expression of this gene as a negative control. We confirmed by *in situ* hybridization that the expression of this gene was not affected in *Foxd* morphants (S1F and S1G Fig).

Our results showed that *Foxd* represses *Prdm1-r*.*a*, *Prdm1-r*.*b*, *Dmrt1*, and *Otx* expression in vegetal cells at the 32-cell stage, although *Otx* is expressed in the posterior vegetal cells of normal embryos and *Foxd* morphants. In addition, *Dlx*.*b*, which is expressed in the entire animal hemisphere, is known to be regulated negatively by Foxd [19], although this gene was not identified to be downstream of *Foxd* in our RNA-seq experiment (Fig 2); this is probably because the number of cells with ectopic *Dlx*.*b* expression is much smaller than the number of animal hemisphere cells with *Dlx*.*b* expression. Because *Prdm1-r*.*a*, *Prdm1-r*.*b*, *Dmrt1*, *Otx*, and *Dlx*.*b* play essential roles in the specification of epidermal and neural fates [11, 19, 20, 22–24, 29], Foxd is likely to suppress ectodermal fates in the vegetal hemisphere.

### A putative Foxd binding site within the *Dmrt1* upstream sequence was important for suppressing ectopic expression in the vegetal hemisphere

To understand the mechanism by which *Foxd* negatively regulates ectodermal fates, we analyzed the upstream regulatory sequence of *Dmrt1* by introducing *lacZ* reporter constructs using electroporation. Experimental embryos were fixed at the 32-cell stage, and reporter expression was examined by *in situ* hybridization.

**Fig 5:**
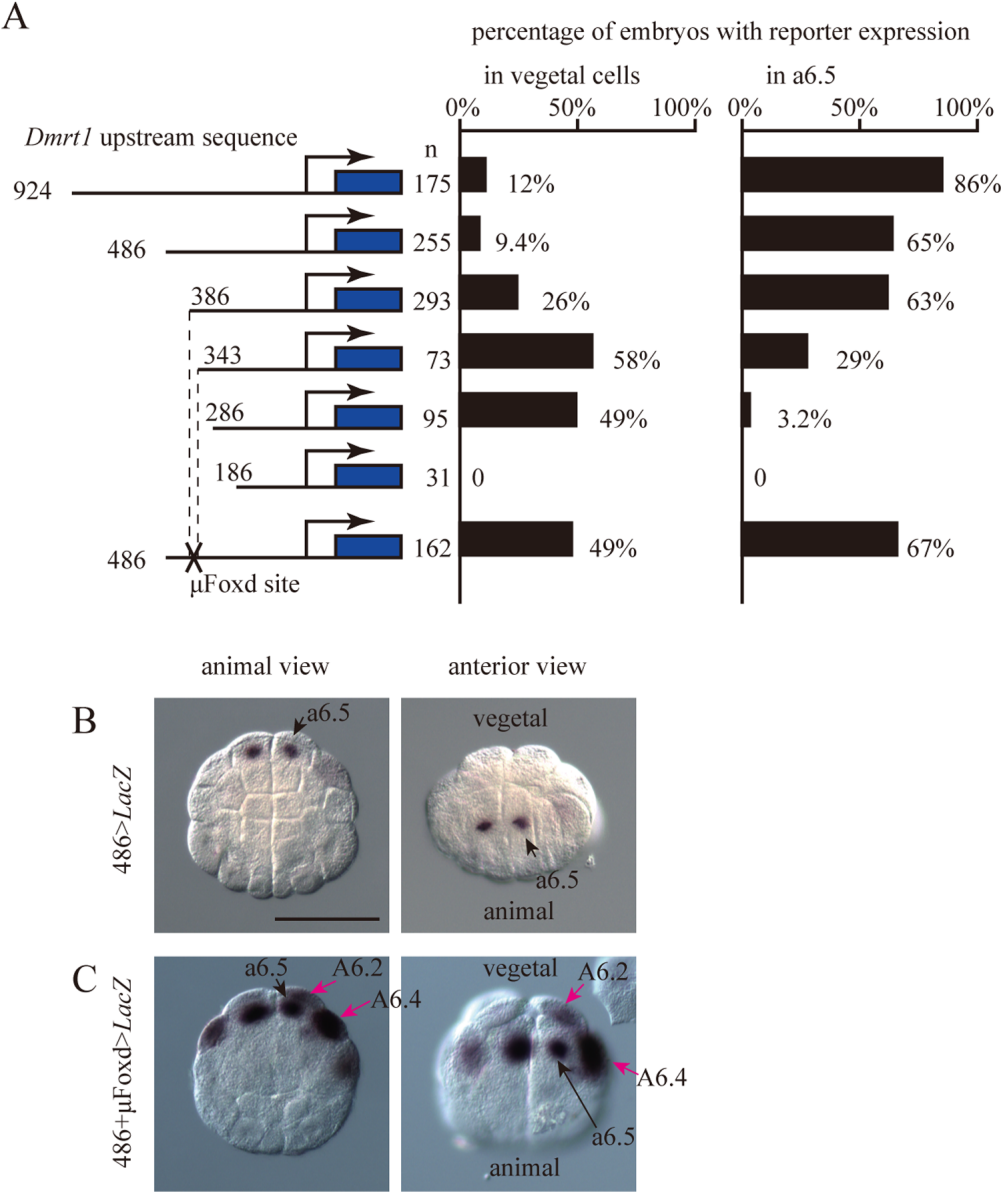
Reporter assay to determine the importance of the Foxd binding site upstream of *Dmrt1*. (A) Analysis of the upstream regulatory region of *Dmrt1*. Illustrations on the left depict the constructs. Blue boxes indicate the *LacZ* gene and SV40 polyadenylation signal. The numbers indicate the relative nucleotide positions from the transcription start site. Mutated Foxd-binding sites are indicated by the letter X. Graphs show the percentage of embryos expressing the reporter in vegetal cells and a6.5 blastomeres. Note that not all cells or embryos could express the reporter because of mosaic incorporation of the electroporated plasmid. (B, C) *LacZ* mRNA expression in embryos electroporated with the constructs containing the 486-bp long upstream region of *Dmrt1* with an intact (B) or mutant (C) Fox binding site. Black arrows indicate normal expression in a6.5, while magenta arrows indicate ectopic expression in *Foxd* morphant embryos. Scale bar, 100 μm.

The 924-base pair (bp) upstream sequence of *Dmrt1*, which was slightly longer than the sequence used in previous studies [29, 30], drove reporter expression specifically in anterior neural cells (a6.5) at the 32-cell stage (Fig 5A; S2 Fig). A construct containing the 486-bp upstream sequence showed almost the same activity (Fig 5A and 5B). Ectopic expression in vegetal cells was increased in constructs containing 386-, 343-, and 286-bp upstream regions, while expression in the a6.5 neural lineage was decreased in the constructs containing 343-, and 286-bp upstream regions. The construct containing the 186-bp upstream sequence did not drive reporter expression. This observation indicated that cis-elements important for expression in the animal hemisphere are present between bases -286 and -386, and that *cis*-elements important for repression in the vegetal hemisphere are present between bases -343 and -386.

We searched candidate Foxd binding sites using the Patser program [31] and a position weight matrix for human FOXD2 [32], which identified one putative Foxd binding site between -343 and -386 (S3 Fig). This site was conserved in the genome of the closely related species *Ciona savignyi* (S3 Fig). Therefore, we mutated this putative binding site. The mutant upstream sequence drove reporter expression in the vegetal hemisphere (Fig 5A and 5C), suggesting that Foxd directly represses *Dmrt1* expression via this site.

### Foxd directly bound to the upstream region of *Dmrt1* and other animal hemisphere-specific genes

**Fig 6:**
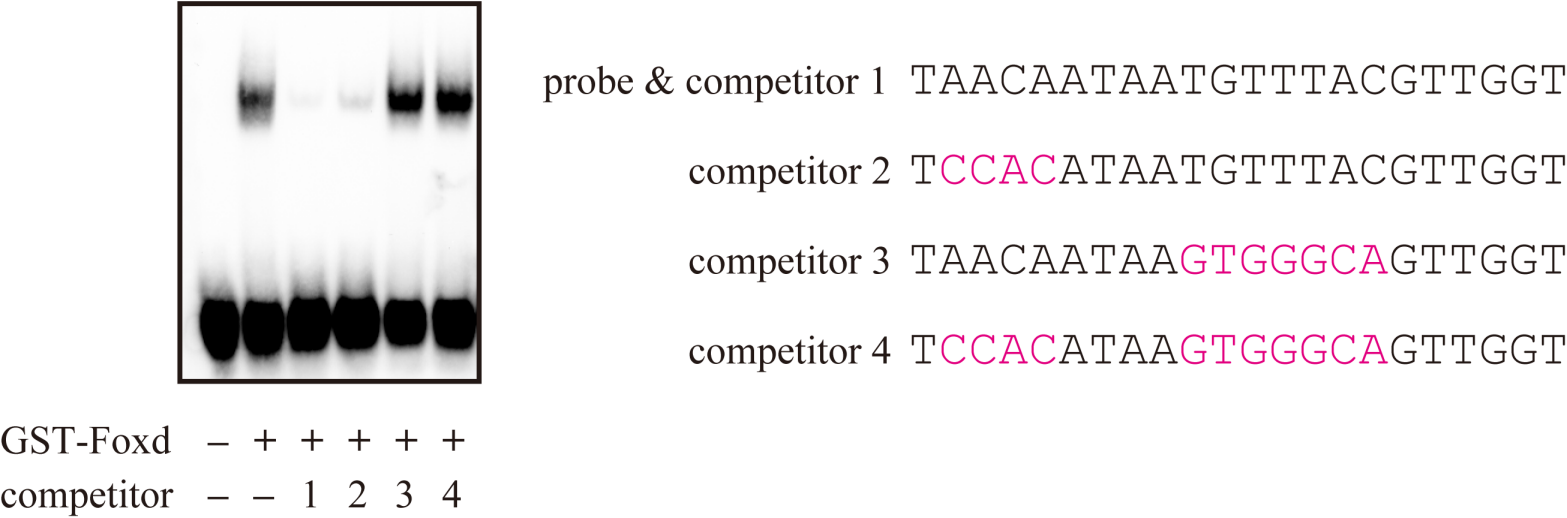
Gel-shift assay to determine Foxd binding to the putative Fox binding site upstream of *Dmrt1*. Foxd.b-GST fusion protein produced in *E. coli* were incubated with a probe containing a putative Fox binding site (TGTTTAC). The shifted band disappeared by co-incubation with competitors 1 and 2, in which the Fox binding site was intact, but not by co-incubation with competitors 3 and 4, in which the Fox binding site was mutated. Sequences of the probe and competitors are shown on the right. Mutated nucleotides are shown in magenta. In competitors 2 and 4, a secondary site (AACA) similar to the primary site was mutated.

To confirm if the identified site could bind Foxd, we performed gel-shift assays (Fig 6). Foxd binding was observed as a shifted band that disappeared upon incubation with a specific competitor (competitor 1) but did not disappear upon incubation with competitors containing a mutation in the putative Fox binding site (competitors 3 and 4). Because the gel-shift probe contained an additional sequence similar to the Fox binding site (AACA), we tested whether this sequence also bound Foxd. The competitor containing a mutation in this second site (competitor 2) did not compete, suggesting that this site does not bind Foxd efficiently.

**Fig 7:**
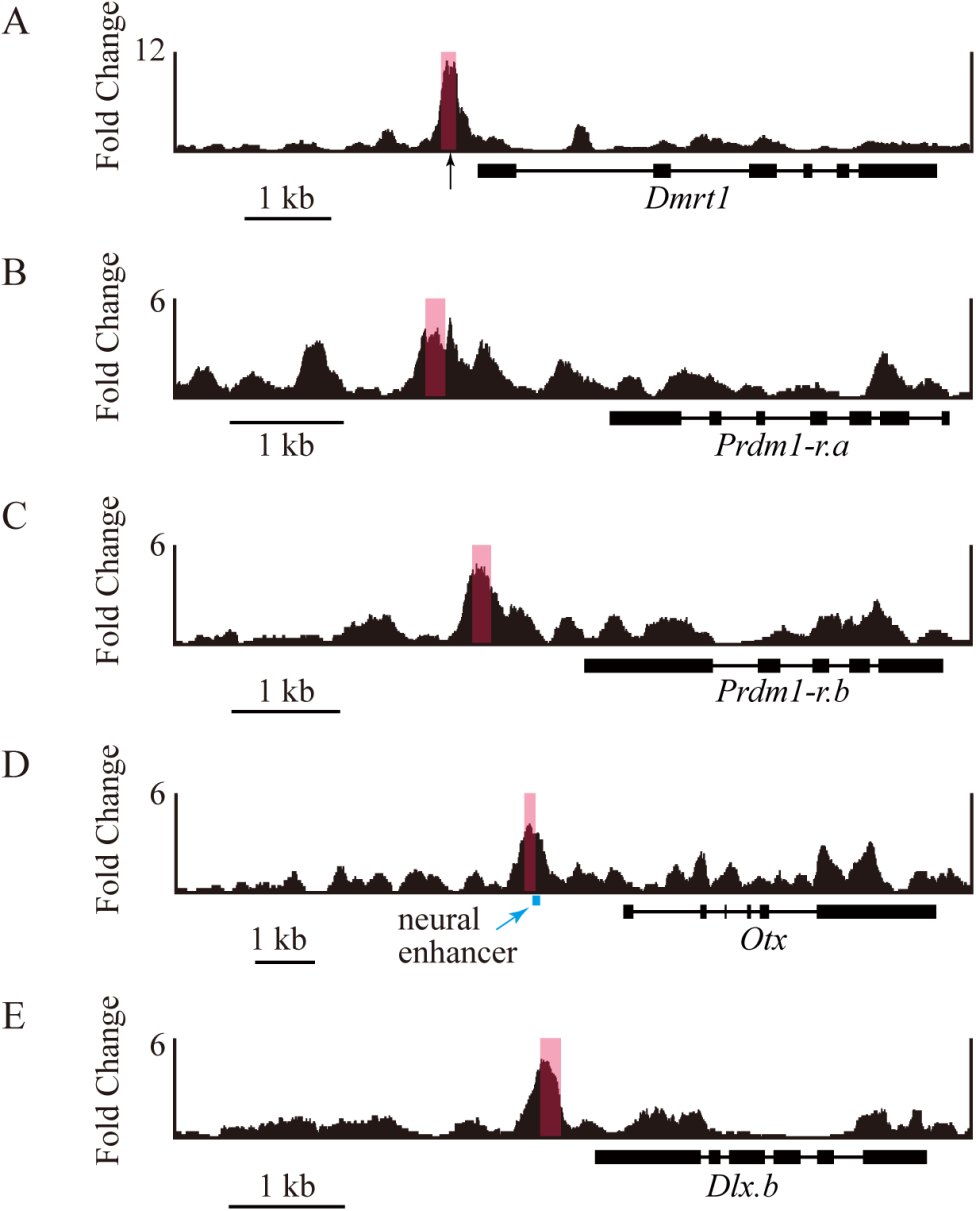
A chromatin-immunoprecipitation assay to determine Foxd binding to the upstream regions of genes that are negatively regulated by Foxd. A construct driving expression of a gene encoding a Foxd-Gfp fusion protein was introduced by electroporation, and anti-Gfp antibodies were used for chromatin immunoprecipitation. Precipitated DNA fragments were analyzed by deep-sequencing. Chromosomal regions containing the upstream regions and exons of (A) *Dmrt1*, (B) *Prdm1-r*.*a*, (C) *Prdm1-r*.*b*, (D) *Otx*, and (E) *Dlx*.*b* are shown. Peaks were called separately for two biological duplicates, and the graphs include data of two biological duplicates. Pink rectangles indicate peak regions commonly identified by the two different peak caller programs; these peak regions were identified in both of the duplicates. A black arrow in (A) indicates the positions of the Foxd binding site identified in the reporter analysis. A cyan line in (D) indicates the location of the neural enhancer revealed by previous studies [11, 35].

Finally, we performed a chromatin-immunoprecipitation assay followed by high-throughput DNA sequencing (ChIP-seq) to confirm that Foxd bound to the regions containing the above putative Foxd binding site *in vivo* at the 32-cell stage (Fig 7). We electroporated an expression construct encoding a Foxd-Gfp fusion protein under the control of the *Foxd* upstream regulatory sequence, and performed a ChIP assay using 32-cell embryos with an anti-Gfp antibody. Two different computer programs identified 114 and 799 peaks, respectively (false discovery rate < 0.1%), of which 63 peaks were common and considered in the subsequent analysis.

Because *Foxd-Gfp* might be overexpressed above physiological levels, it is possible that the above binding interactions were stronger than interactions that would normally occur in normal embryos. However, among 52,518 of ‘GTAAACA’ sequences found in the genome, only 9 sites were included in the 63 peaks identified by the ChIP-seq assay, suggesting that Foxd-Gfp does not bind non-specifically to all potential binding sites.

As shown in Fig 7A, the upstream region of *Dmrt1* around the Fox binding site identified above bound Foxd, suggesting direct regulation of *Dmrt1* by Foxd. In addition, we found peak regions in the upstream sequences of *Prdm1-r*.*a*, *Prdm1-r*.*b*, *Otx*, and *Dlx*.*b* (Fig 7B–7E). All these peak regions contained Foxd binding motifs that were identifiable by the Patser program [31] and a position weight matrix for human FOXD2 [32], although their scores were less than the scores of *Dmrt1* (S4 Fig). Meanwhile, the 63 significant peaks were not found in the upstream regulatory region of *Tfap2-r*.*b*, which is not regulated by Foxd as described above, although a weak, insignificant peak was observed (S5 Fig). Therefore, it is conceivable that *Dmrt1*, *Prdm1-r*.*a*, *Prdm1-r*.*b*, *Otx*, and *Dlx*.*b* are direct targets of Foxd.

## Discussion

### *Foxd* maintains differential gene expression along the animal-vegetal axis

**Fig 8:**
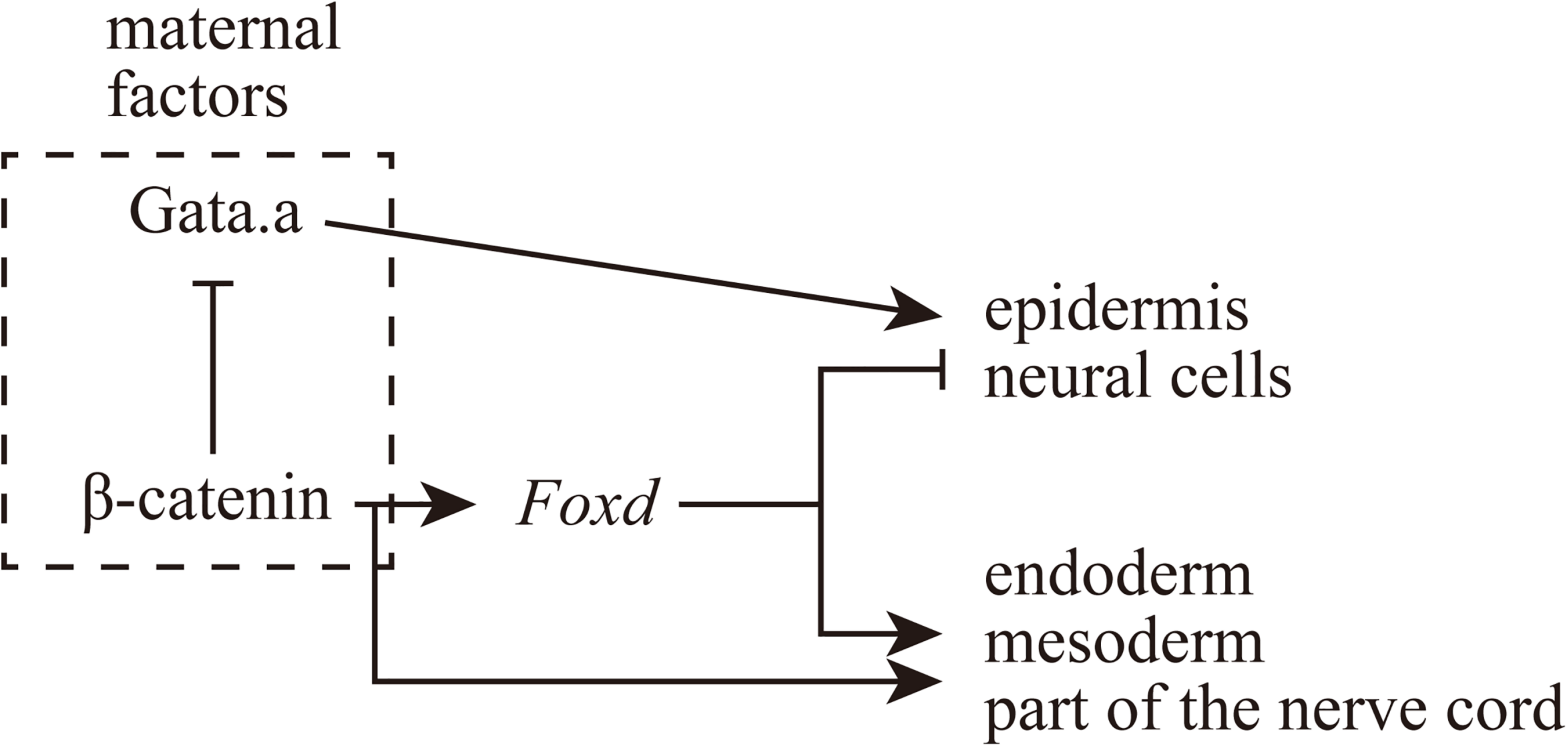
Schematic representation of a gene regulatory circuit essential for determining endomesodermal fate in the vegetal hemisphere. Two domains with different gene expression patterns along the animal-vegetal axis are established initially by maternal β-catenin and Gata.a, and then stabilized by Foxd. These two domains mostly correspond to the ectoderm and endomesoderm.

In ascidian embryos, maternal factors establish differential gene expression patterns between the animal and vegetal hemispheres, which largely correspond to the ectodermal and endomesodermal lineages (with the exception of part of the nerve cord, which is derived from the vegetal hemisphere). Gata.a and β-catenin/Tcf7 activate specific gene expression in these two domains at the 16-cell stage. However, our present results indicated that this segregation between the animal and vegetal hemisphere lineages was not robust enough to maintain this segregation alone. We found that Foxd activity commits vegetal cells to the endomesoderm fate by repressing ectoderm genes including *Prdm1-r*.*a*, *Prdm1-r*.*b*, *Dmrt1*, *Otx*, and *Dlx*.*b* (Fig 8), although Foxd may not necessarily repress all genes that are expressed in the animal hemisphere. In other words, maternal factors generated a transient regulatory stage, which was maintained by Foxd activity. Thus, animal hemisphere gene expression is suppressed in the vegetal hemisphere continuously during early embryogenesis. First, Gata.a activity is suppressed by β-catenin/Tcf7 in the vegetal hemisphere of the 16-cell embryo [15], and then Foxd, which is activated by β-catenin/Tcf7, directly represses animal hemisphere genes in the vegetal hemisphere at the 32-cell stage and thereafter.

In *Foxd* morphants, *Prdm1-r*.*a* and *Prdm1-r*.*b* were activated ectopically in both the anterior and posterior vegetal cells, while *Dmrt1* and *Otx* were activated ectopically only in the anterior cells. Activators for *Dmrt1* might not be present in the posterior vegetal cells. In normal embryos, *Dmrt1* is activated only in the anterior neural cells, because *Foxa*.*a*, which encodes an activator for *Dmrt1*, is not expressed in the posterior neural cells [19, 25, 33]. *Foxa*.*a* expression indeed begins in the anterior half of the 8-cell embryo, although it is expressed in posterior vegetal cells at the 16-cell stage and thereafter [33, 34]. Meanwhile, in normal embryos, *Otx* is expressed in the posterior vegetal cells except the most posterior cells, in addition to the animal neural cells [20]. Different enhancers are responsible for expression in these two regions [11, 35]. Therefore, even if the neural enhancer of *Otx* is ectopically activated in these posterior vegetal cells, this ectopic activation cannot be detected by *in situ* hybridization. Indeed, one of the peak regions in the *Otx* upstream region partly overlaps the neural enhancer identified in previous studies [11, 35] (Fig 7D). Activation of *Otx* in the vegetal hemisphere by different enhancers may explain why we detected differential expression of *Otx* only at the 32-cell stage by the RNA-seq experiments.

In addition to the repressive function shown above, Foxd functions as an activator; it activates *Zic-r*.*b* and *Lhx3/4* cooperatively with *Foxa*.*a* and *Fgf9/16/20* [16, 19, 27]. A ChIP assay showed that Foxd binds to upstream regions of Foxd-regulated genes at the 64-cell stage [36]. Reporter assays also showed that two Fox-binding sites within the upstream sequence of *Zic-r*.*b*, which is activated by Foxd, are essential for its expression [37]. *Lhx3/4* is also likely to be a direct target of Foxd, because *Lhx3/4* is expressed at the 32-cell stage under the control of *Foxd* [16], and because Foxd is bound to the upstream region of *Lhx3/4* at the 64-cell stage [36]. Thus, Foxd is a dual-functional protein; it simultaneously promotes endomesodermal fates and inhibits ectodermal fates.

It has been proposed that there are sub-circuits responsible for locking down regulatory states [38]. In *Ciona* early embryos, Foxd maintains the regulatory state of the vegetal hemisphere, and therefore this dual-functional protein may alone work like such a sub-circuit to lock down dynamic states.

### Foxd works as a transcriptional activator and repressor

In *Xenopus*, FoxD4L1.1 has a dual role as a transcriptional activator and repressor in the neural ectoderm; it activates genes that keep cells in a proliferative state and represses genes that promote differentiation [39]. *Xenopus* FoxD4L1.1 is also involved in repressing BMP signaling, thereby suppressing epidermal fate. The activating function is mediated in *Xenopus* by an acidic domain near the N-terminus and the repressing function at least partly depends on an Engrailed homology region-1 (Eh-1) located in the C-terminal region. *Ciona* Foxd also contains a putative acidic domain near the N-terminus and an Eh-1 motif in the C-terminal region (S6 Fig). In both *Ciona* and *Xenopus*, *Foxd* acts as a robust binary switch that promotes one fate and suppresses the other fate. This might be an evolutionarily conserved function of Foxd.

A previous study identified two critical Fox-binding sites to which Foxd might bind in the upstream region of *Zic-r*.*b* [37]. The sequences of these sites are slightly different from the sequence of the Foxd-binding site for *Dmrt1* and those found in the peak regions in the upstream regions of *Prdm1-r*.*a*, *Prdm1-r*.*b*, *Otx*, and *Dlx*.*b*. In the ChIP-seq assay of the present study, we did not find clear binding peaks upstream of *Zic-r*.*b*, although our previous ChIP-chip assay using slightly older embryos exhibited peaks [36]. Therefore, the binding sites in the upstream regions of *Prdm1-r*.*a*, *Prdm1-r*.*b*, *Otx*, and *Dlx*.*b* might be stronger than the binding sites upstream of *Zic-r*.*b*. Indeed, at least one Foxd binding motif in each of the peak regions in the upstream regions of *Prdm1-r*.*a*, *Prdm1-r*.*b*, *Otx*, and *Dlx*.*b* gave a higher score than the Fox binding sites found in *Zic-r*.*b* (S4B Fig). Such a qualitative difference might be important for Foxd to work as an activator or a repressor. In the ascidian embryo, Sox1/2/3, and Gata.a are important for specification of ectodermal fate [11, 12, 15, 23]. Because there are clear Sox and Gata binding motifs in the peak regions of *Dmrt1*, *Prdm1-r*.*a*, *Prdm1-r*.*b*, *Otx*, and *Dlx*.*b*, it is possible that Sox1/2/3 and Gata.a help Foxd to act as a repressor.

## Materials and methods

### Animals, whole-mount *in situ* hybridization, and gene identifiers

*Ciona intestinalis* (type A; this type is also called *Ciona robusta*) adults were obtained from the National Bio-Resource Project for *Ciona*. cDNA clones were obtained from our EST clone collection [40]. Whole-mount *in situ* hybridization was performed as described previously [25]. Identifiers for genes examined in the present study are shown in S1 Table, according to the nomenclature rule proposed in a recent paper [41].

### Gene knockdown, overexpression and reporter assays

A morpholino oligonucleotide (MO; Gene Tools, LLC) for *Foxd* knock-down is designed to block translation of two paralogous *Foxd* genes, *Foxd*.*a* and *Foxd*.*b* (5′-GCACACAACACTGCACTGTCATCAT-3′). This MO has been used previously, and its specificity has been evaluated [19, 25]. The MO was introduced by microinjection under a microscope.

The coding sequence of *Foxd*.*b* was cloned into pBluscript RN3 [42], and *Foxd* mRNA was transcribed using the mMESSAGE mMACHINE T3 Transcription Kit (Life technologies).

Reporter constructs were introduced into fertilized eggs by electroporation. Chromosomal positions of the upstream sequences for reporter constructs and the mutated sequence are indicated in S2 Fig. We randomly chose embryos introduced with reporter constructs to examine reporter construct expression by *in situ* hybridization.

We performed all gene knockdown experiments and reporter gene assays at least twice with different batches of embryos.

### Gel-shift assay

Recombinant Foxd.b protein was produced as a fusion protein of the Foxd DNA-binding domain and glutathione S-transferase in *Escherichia coli* BL21 star DE3 strain (Thermo Fisher Scientific), and the protein was purified under a native condition using glutathione Sepharose 4B (GE Healthcare). After annealing two complementary oligonucleotides (5’-AAATAACAATAATGTTTACGTTGGT-3’ and 5’-AAAACCAACGTAAACATTATTGTTA-3’), both protruding ends of the double-stranded oligonucleotides were filled with biotin-11-dUTP, and this biotin-labelled oligonucleotide was used as a probe. Proteins and the biotin-labeled probe were mixed in 10 mM Tris (pH 7.5), 50 mM KCl, 1 mM DTT, 1 mM EDTA, 50 ng/μL poly(dIdC), 2.5% glycerol, and 0.05% NP40 with or without competitor double-stranded DNAs (a 100 fold molar excess) shown in Fig 6. Proteins amounts were empirically determined. Protein–DNA complexes were detected using an AP-conjugated anti-biotin antibody (Roche) and CDP-star substrate (Roche).

### RNA sequencing (RNA-seq)

For RNA-seq experiments, 50 unperturbed and Foxd-morphant embryos were collected at the 32-, 64-, and 112-cell stages. RNA was extracted using a Dynabeads mRNA DIRECT Purification Kit (Thermo Fischer Scientific) and libraries were made with an Ion Total RNA-Seq kit ver 2 (Thermo Fischer Scientific). The libraries were sequenced with an Ion PGM instrument (Thermo Fischer Scientific) (SRA accession number: DRA005206). We did not utilize duplicates because we used this experiment for screening purposes, and the obtained results were confirmed using other methods, as explained in the Results section. NOISeq [26] was used to identify differentially expressed genes.

### Chromatin immunoprecipitation

We used a DNA construct encoding GFP-tagged Foxd under the control of the *Foxd* promoter [36]. Embryos were fixed at the 32-cell stage. The embryos were subjected to ChIP analysis using anti-GFP antibodies, and the immunoprecipitated DNA was amplified by ligation-mediated PCR [36]. Whole cell extract DNA was used as a control. Then, high-throughput DNA sequencing was performed with the Ion PGM instrument (SRA accession number: DRA005285). To identify peak regions, we used two different programs called Homer [43] with options “-style factor -F 4 -P 0.01 -L 4 -localSize 3000” and MACS2 [44] with an option “--nomodel -q 0.001".

### RT-qPCR

For RT-qPCR, we extracted RNA from wild-type embryos and embryos injected with *Foxd* mRNA. The RNA was converted to cDNA by the Cells-to-Ct kit (Thermo Fisher Scientific). The obtained cDNA samples were then analyzed by quantitative PCR with the SYBR green method. For each qPCR, the amount of cDNA used was equivalent to two-thirds of an embryo. Primers used were: *Dmrt1*, 5’-CGCTGAACGACAACGAGTCAT-3’ and 5’-TTCGTTTTCCTCTTGTGCTTGTT-3’; *Foxd*.*a*, 5’-AGTTTCTTCCCCACAGTTCCAA-3’ and 5’-GGTTTGTTGTATCCGGGATGTT-3’; *Foxd*.*b*, 5’-GCAGTACGCATTCCGCAAT-3’ and 5’-CGGAACAAAAACACAAAAGTCAAA-3’; *Pou2*, 5’- AAGATGGTTGCTGGATGCTAATAAT-3’ and 5’-TTGGATTGGAGTGGGAATAACAA-3’.

### Ethics statement

*Ciona intestinalis* is excluded from legislation regulating scientific research on animals in Japan. Although there is no scientific evidence that *Ciona intestinalis* can experience pain, discomfort or stress, we made our best efforts to minimize potential harm that *Ciona* individuals might experience when we obtained eggs and sperm from them.

## Supporting information

S1 Fig Expression of *Foxd*, *Fgf9/16/20*, and *Tfap2-r*.*b* in *Foxd* morphants. (A, B) The expression of *Foxd* revealed by *in situ* hybridization in (A) control and (B) *Foxd* morphants at the 64-cell stage. (C) The amount of endogenous *Foxd* mRNA was measured by RT-qPCR in uninjected control embryos and embryos injected with 2.3 pg of *Foxd* mRNA. The relative amount of mRNA in the experimental embryos compared with control embryos is shown. A maternal mRNA, *Pou2*, was used as an endogenous control. Error bars indicate mean±s.d. between two technical duplicates. The results of two independent experiments are shown in different colors. (D–G) The expression of (D, E) *Fgf9/16/20*, and (F, G) *Tfap2-r*.*b* revealed by *in situ* hybridization in (D, F) control unperturbed embryos and (E, G) *Foxd* morphant embryos at the 16-cell (D, E) and 32-cell (F, G) stages. Note that *Foxd* expression was not downregulated in the vegetal hemisphere of *Foxd* morphants (A, B) and that the expression of *Fgf9/16/20* and *Tfap2-r*.*b* was not changed (D–G), although *Fgf9/16/20* expression is downregulated in later embryos (Fig 2) [19]. The number of morphant embryos examined and the proportion of embryos that each panel represents are shown within the panels. Scale bar, 100 μm.
(TIF)

S2 Fig Upstream sequence of *Dmrt1*. The numbers indicate the relative nucleotide positions from the transcriptional start site. The sequence of the fragment used for the gel-shift assay in Fig 6 is underlined, and the putative Foxd site is indicated in cyan. The mutation introduced in the Foxd site is shown in magenta.
(TIF)

S3 Fig An alignment of the upstream sequences of *Dmrt1* of *C*. *intestinalis* and *C*. *savignyi*. (A) Asterisks indicate conserved nucleotides. T-Coffee [47] was used for generating this alignment. Putative Foxd binding sites, which were identified by Patser [31], are shown in cyan with scores. (B) A consensus sequence for human FOXD2 [32], which was used for identifying the putative Foxd binding sites, is shown as a sequence logo [48].
(TIF)

S4 Fig Foxd-binding regions identified by chromatin-immunoprecipitation. (A) Nucleotide sequences of the Foxd-binding regions in the upstream regions of *Dmrt1*, *Prdm1-r*.*a*, *Prdm1-r*.*b*, *Otx*, and *Dlx*.*b*, which were identified by the chromatin-immunoprecipitation and are shown in pink boxes in Fig 7. Putative Foxd-binding sites are shown in magenta. (B) An alignment of the putative Foxd binding sites found in (A) and those in the *Zic-r*.*b* (*ZicL*) upstream region identified previously [37]. Scores on the right were calculated by the Patser program and a position weight matrix for human FOXD2 binding sites [32], which is represented in S3B Fig.
(TIF)

S5 Fig A chromatin-immunoprecipitation assay to determine Foxd binding to the upstream region of *Tfap2-r*.*b*. Because *Tfap2-r*.*b* was not regulated by *Foxd* (S1F and S1G Fig), the upstream region of *Tfap2-r*.*b* is shown as a negative control for genes shown in Fig 7. Significant peaks were not identified by the computer programs in this genomic region. The graphs include data of two biological duplicates.
(TIF)

S6 Fig An alignment of Foxd proteins of *C*. *intestinalis* and *Xenopus laevis*. Conserved and similar amino acids are shown by black and gray boxes, respectively. The forkhead domains, Eh-1 domains, and putative acidic domains are enclosed by black lines, and acidic amino acids in the putative acidic region of the N-terminal half are shown in magenta.
(TIF)

S1 Table Gene identifiers. (DOCX)
