# Supplementary figures and images for: Differential gene expression along the animal-vegetal axis in the ascidian embryo is maintained by a dual functional protein Foxd
Source: PLoS Genet. 2017 May 17;13(5):e1006741. doi: 10.1371/journal.pgen.1006741 (PMC5453608; doi:10.1371/journal.pgen.1006741)

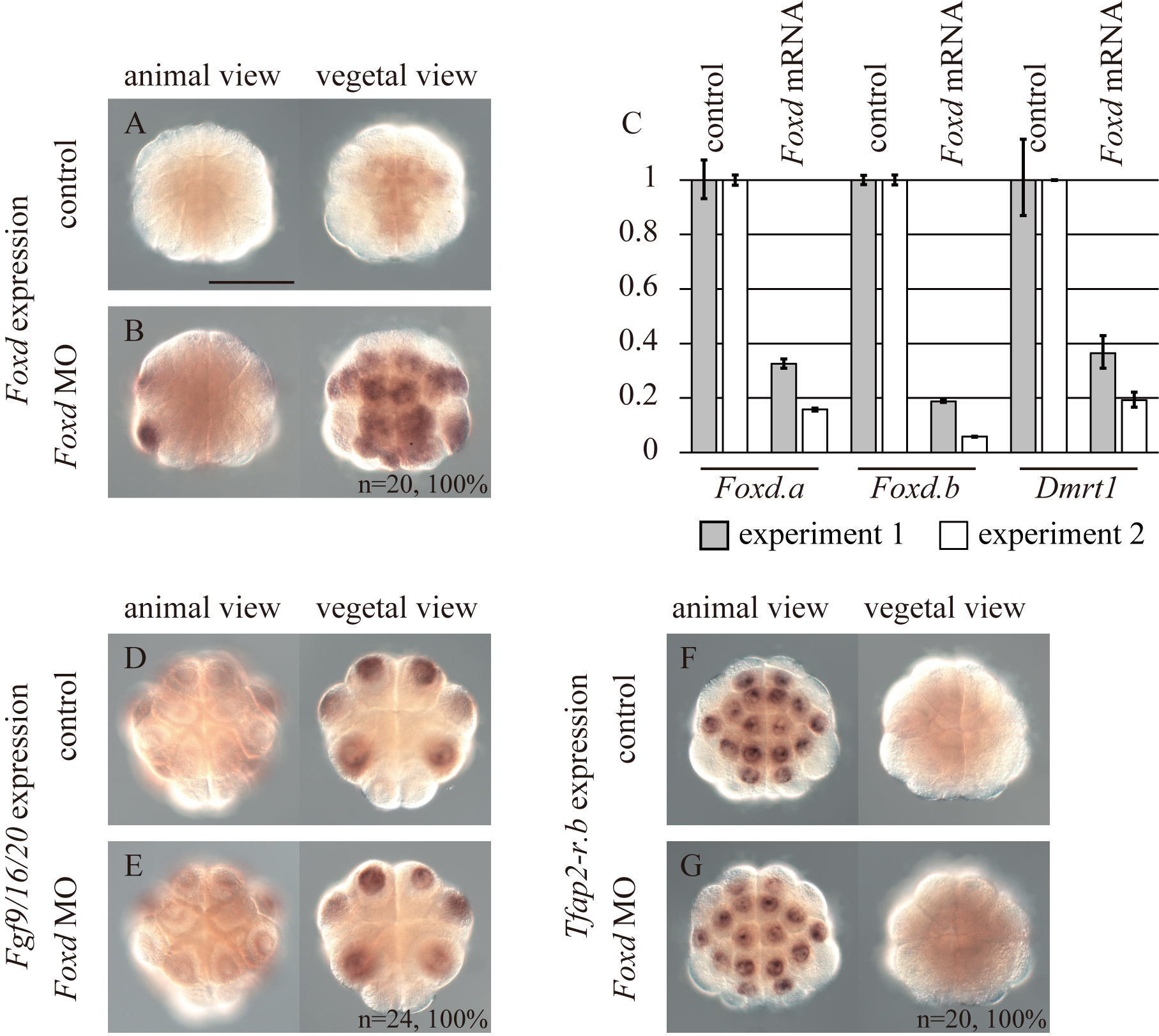

Supplement: S1 Fig — (A, B) The expression of Foxd revealed by in situ hybridization in (A) control and (B) Foxd morphants at the 64-cell stage. (C) The amount of endogenous Foxd mRNA was measured by RT-qPCR in uninjected control embryos and embryos injected with 2.3 pg of Foxd mRNA. The relative amount of mRNA in the experimental embryos compared with control embryos is shown. A maternal mRNA, Pou2, was used as an endogenous control. Error bars indicate mean±s.d. between two technical duplicates. The results of two independent experiments are shown in different colors. (D–G) The expression of (D, E) Fgf9/16/20, and (F, G) Tfap2-r.b revealed by in situ hybridization in (D, F) control unperturbed embryos and (E, G) Foxd morphant embryos at the 16-cell (D, E) and 32-cell (F, G) stages. Note that Foxd expression was not downregulated in the vegetal hemisphere of Foxd morphants (A, B) and that the expression of Fgf9/16/20 and Tfap2-r.b was not changed (D–G), although Fgf9/16/20 expression is downregulated in later embryos (Fig 2) [19]. The number of morphant embryos examined and the proportion of embryos that each panel represents are shown within the panels. Scale bar, 100 μm. (TIF) [file pgen.1006741.s001.tif]

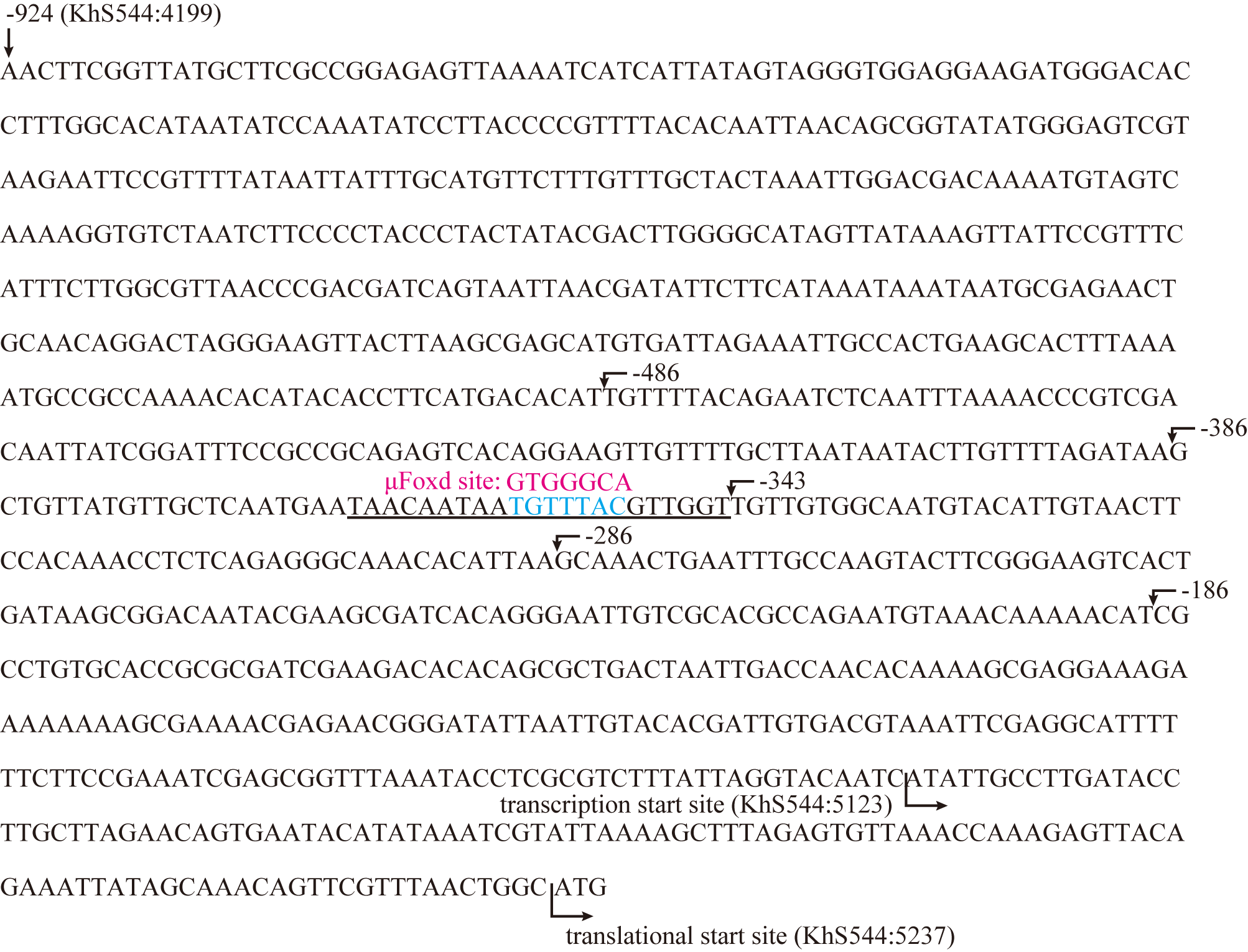

Supplement: S2 Fig — The numbers indicate the relative nucleotide positions from the transcriptional start site. The sequence of the fragment used for the gel-shift assay in Fig 6 is underlined, and the putative Foxd site is indicated in cyan. The mutation introduced in the Foxd site is shown in magenta. (TIF) [file pgen.1006741.s002.tif]

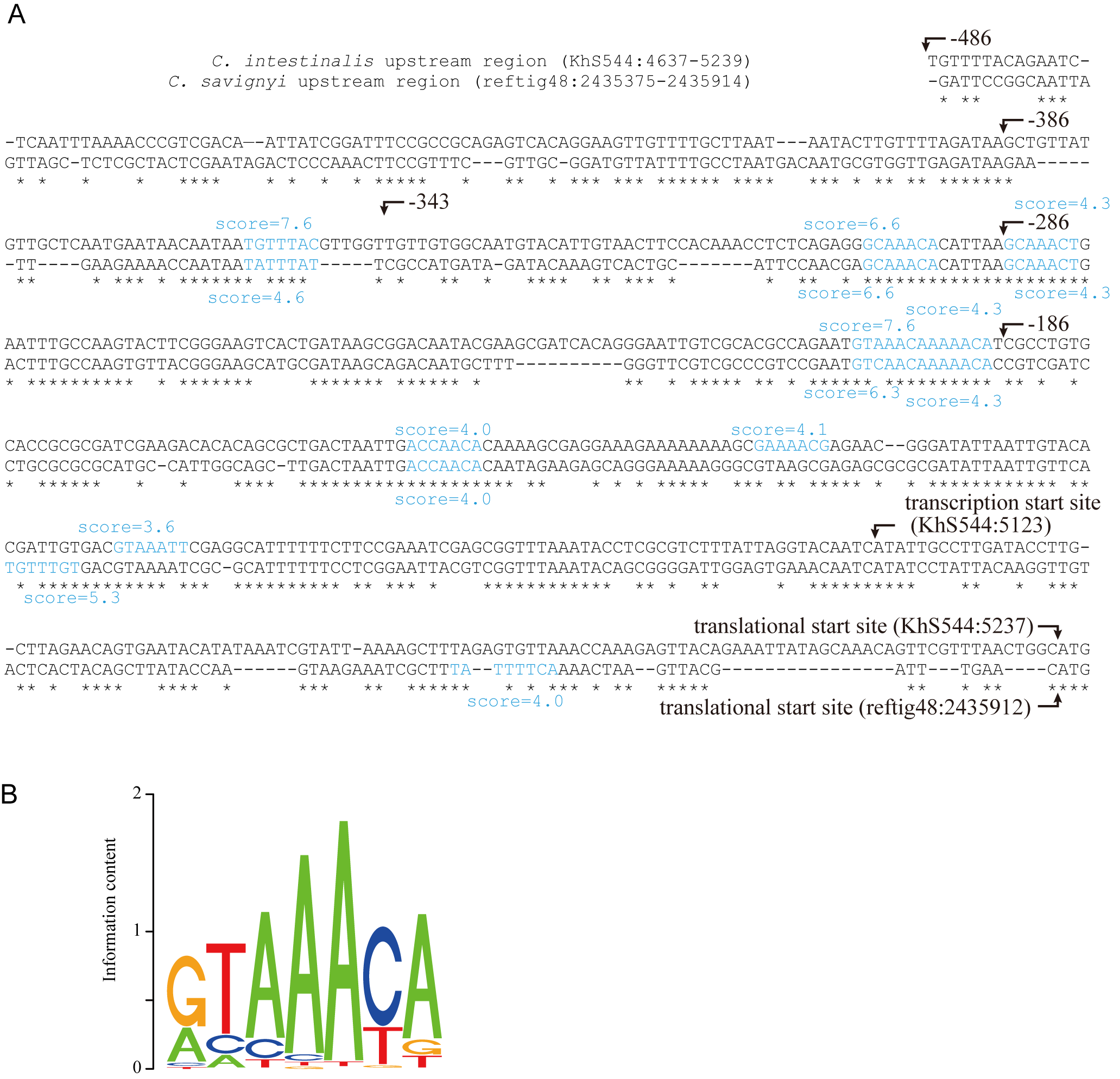

Supplement: S3 Fig — (A) Asterisks indicate conserved nucleotides. T-Coffee [47] was used for generating this alignment. Putative Foxd binding sites, which were identified by Patser [31], are shown in cyan with scores. (B) A consensus sequence for human FOXD2 [32], which was used for identifying the putative Foxd binding sites, is shown as a sequence logo [48]. (TIF) [file pgen.1006741.s003.tif]

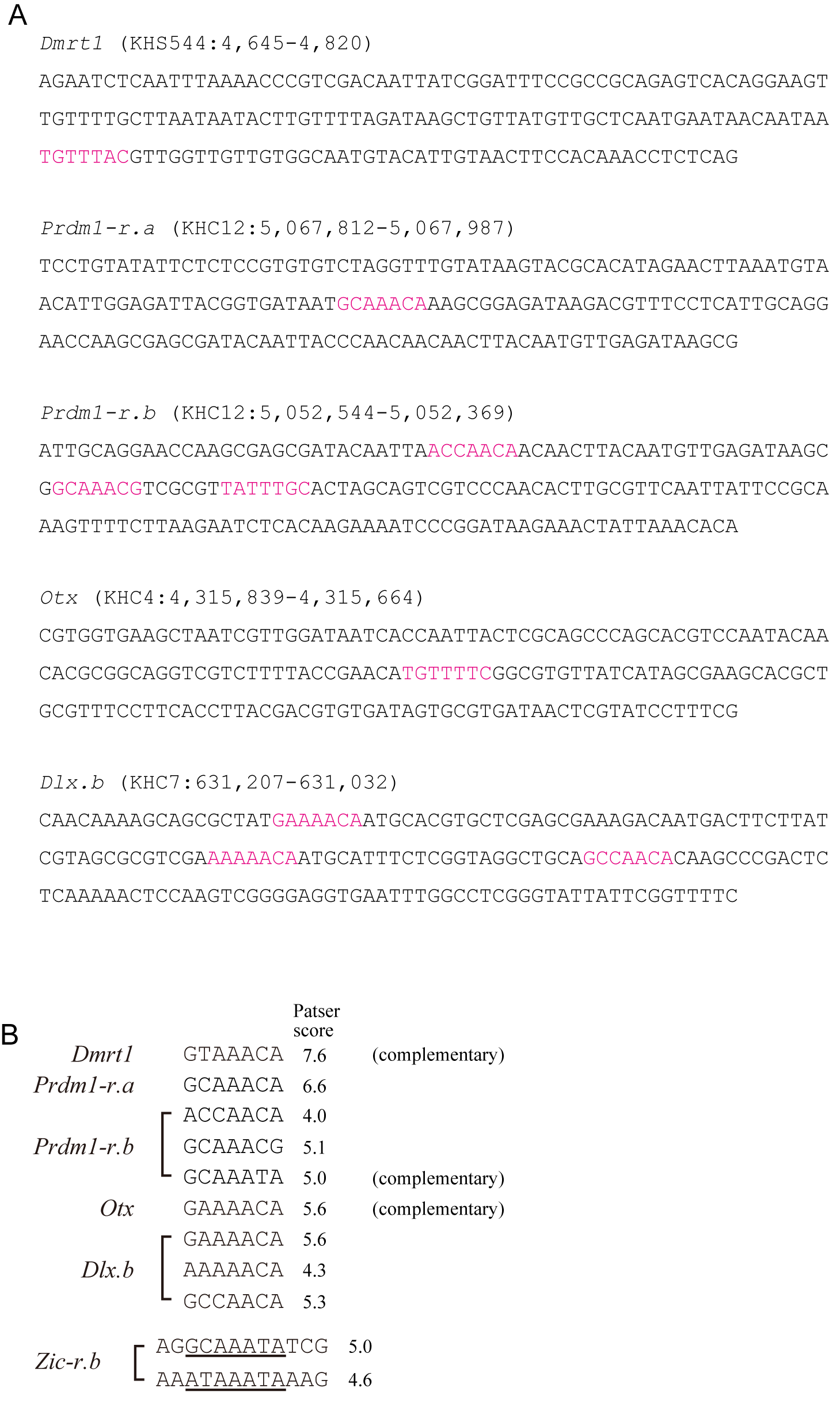

Supplement: S4 Fig — (A) Nucleotide sequences of the Foxd-binding regions in the upstream regions of Dmrt1, Prdm1-r.a, Prdm1-r.b, Otx, and Dlx.b, which were identified by the chromatin-immunoprecipitation and are shown in pink boxes in Fig 7. Putative Foxd-binding sites are shown in magenta. (B) An alignment of the putative Foxd binding sites found in (A) and those in the Zic-r.b (ZicL) upstream region identified previously [37]. Scores on the right were calculated by the Patser program and a position weight matrix for human FOXD2 binding sites [32], which is represented in S3B Fig. (TIF) [file pgen.1006741.s004.tif]

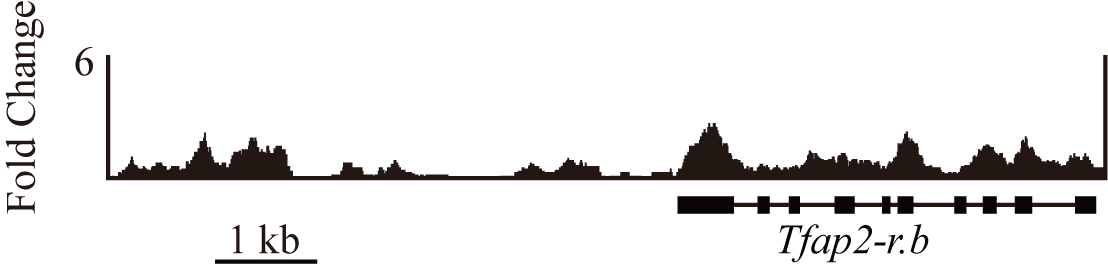

Supplement: S5 Fig — Because Tfap2-r.b was not regulated by Foxd (S1F and S1G Fig), the upstream region of Tfap2-r.b is shown as a negative control for genes shown in Fig 7. Significant peaks were not identified by the computer programs in this genomic region. The graphs include data of two biological duplicates. (TIF) [file pgen.1006741.s005.tif]

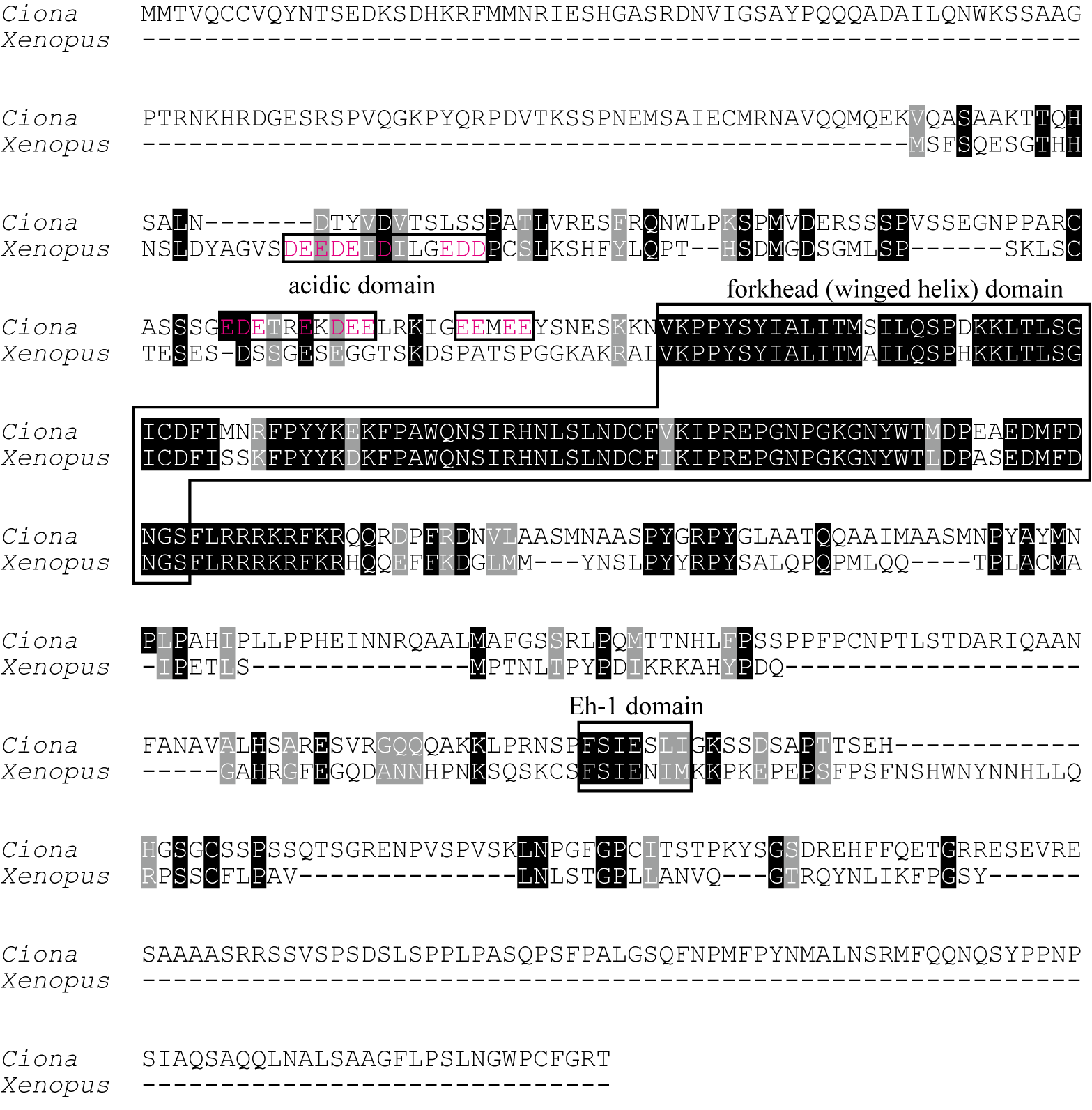

Supplement: S6 Fig — Conserved and similar amino acids are shown by black and gray boxes, respectively. The forkhead domains, Eh-1 domains, and putative acidic domains are enclosed by black lines, and acidic amino acids in the putative acidic region of the N-terminal half are shown in magenta. (TIF) [file pgen.1006741.s006.tif]
