# Supplementary material for: Differential gene expression along the animal-vegetal axis in the ascidian embryo is maintained by a dual functional protein Foxd
Source: PLoS Genet. 2017 May 17;13(5):e1006741. doi: 10.1371/journal.pgen.1006741 (PMC5453608; doi:10.1371/journal.pgen.1006741)
Supplement: S1 Table — (DOCX) [file pgen.1006741.s007.docx]

**S1 Table. Gene identifiers**

| Genes | Identifiers |
| --- | --- |
| *β-catenin (Cttnb)* | CG.KH2012.C9.53 |
| *Brachyury (T)* | CG.KH2012.S1404.1 |
| *Dlx.b* | CG.KH2012.C7.243 |
| *Dmrt-1 (Dmrt.a)* | CG.KH2012.S544.3 |
| *Efna.d* | CG.KH2012.C3.716 |
| *Fgf8/17/18* | CG.KH2012.C5.5 |
| *Fgf9/16/20* | CG.KH2012.C2.125 |
| *Foxa.a* | CG.KH2012.C11.313 |
| *Foxb* | CG.KH2012.C4.341 |
| *Foxd.a* | CG.KH2012.C8.890 |
| *Foxd.b* | CG.KH2012.C8.396 |
| *Gata.a* | CG.KH2012.L20.1 |
| *Lhx3/4* | CG.KH2012.S215.4 |
| *Mnx* | CG.KH2012.L128.12 |
| *Nkx2-1 (Ttf1)* | CG.KH2012.C10.338 |
| *Otx* | CG.KH2012.C4.84 |
| *Prdm1-r.a* | CG.KH2012.C12.493 |
| *Prdm1-r.b* | CG.KH2012.C12.105 |
| *Tcf7* | CG.KH2012.C6.71 |
| *Tfap2-r.b* | CG.KH2012.C7.43 |
| *Pou2* | CG.KH2012.C4.85 |
